# Bioactive Potential of *Origanum vulgare* Rhizomes: Phenolic Composition, Antioxidant, Antibacterial, and Cytotoxicity Profiles

**DOI:** 10.1002/fsn3.71413

**Published:** 2026-01-09

**Authors:** Elena Kurin, Kamila Dokupilová, Ema Kostovčíková, Lívia Slobodníková, Eva Drobná, Iveta Čičová, Veronika Brindza Lachová, Jana Sabová, Peter Gál, Milan Nagy, Pavel Mučaji, Silvia Bittner Fialová

**Affiliations:** ^1^ Department of Pharmacognosy and Botany, Faculty of Pharmacy Comenius University Bratislava Bratislava Slovakia; ^2^ Institute of Microbiology, Faculty of Medicine Comenius University Bratislava, and the University Hospital in Bratislava Bratislava Slovakia; ^3^ Department of Cell and Molecular Biology of Drugs, Faculty of Pharmacy Comenius University in Bratislava Bratislava Slovakia; ^4^ National Agricultural and Food Centre Research Institute of Plant Production Piešťany Slovakia; ^5^ Department of Pharmacology, Faculty of Medicine Pavol Jozef Šafárik University in Košice Košice Slovakia; ^6^ Prague Burn Centre Third Faculty of Medicine and University Hospital Královské Vinohrady Prague Czech Republic

**Keywords:** antibacterial, antioxidant, leaf, microcalorimetry, *Origanum*, rhizome

## Abstract

Utilization of plant by‐products contributes to the efficient use of natural resources and expands the range of bioactive materials of botanical origin. Oregano ( *Origanum vulgare* L.) is widely recognized for its active metabolites; however, its underground parts have received little attention. In this study, 14 phenolic compounds were identified in water extracts of oregano leaves (OVL) and rhizomes (OVR) using LC–MS/MS‐DAD. Both extracts contained rosmarinic acid, oreganol C, and caffeic acid, while OVL also included oreganol A, oreganol B, and luteolin‐7‐*O*‐diglucuronide. Remarkably, OVR was revealed as a previously unreported natural source of salvianolic acid A. The antioxidant potential, evaluated by DPPH, ABTS, and H_2_O_2_ scavenging assays, demonstrated comparable activities of OVL and OVR. Antimicrobial testing (broth microdilution and isothermal microcalorimetry) confirmed inhibitory effects against *Staphylococcus aureus* (MSSA, MRSA), *Proteus mirabilis* , and *Enterococcus faecalis* . Notably, OVR showed stronger inhibition of staphylococcal metabolic activity (≥ 0.75 mg/mL) than OVL (≥ 5.0 mg/mL). Cytotoxicity assessed by MTT assay on HaCaT keratinocytes indicated no significant reduction in cell viability at concentrations ≤ 100 μg/mL, supporting the biocompatibility of both extracts. The rhizome extract further showed a safety profile comparable to that of the leaf extract, supporting its potential for further biomedical use. The present study demonstrates that oregano rhizomes represent an unexplored botanical source of antioxidant and anti‐staphylococcal compounds, expanding current knowledge on the phytochemical potential of this species.

## Graphical abstract

Oregano rhizomes, often treated as waste, contain valuable bioactive compounds, highlighting their sustainable potential. LC–MS/MS‐DAD analysis identified 14 phenolic compounds, with salvianolic acid A unique to rhizomes. Rhizome water extracts showed strong antioxidant activity and notable antimicrobial effects, effectively inhibiting MRSA and MSSA.

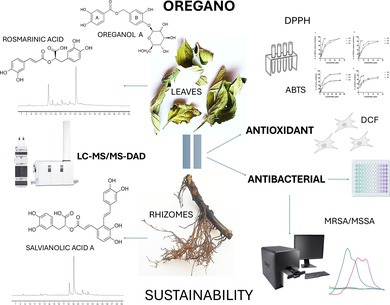

**Abbreviations**
ABTS: 2,2‐diphenyl‐1‐picrylhydrazyl radical
ATCC: American Type Culture Collection
ATM: antimicrobial
CCM: Czech Collection of Microorganisms
DCF: 2′,7′‐dichlorofluorescein
DPPH: 2,2‐diphenyl‐1‐picrylhydrazyl
HaCaT: human epidermal keratinocytes
IMC: isothermal microcalorimetry
LC–MS/MS‐DAD: liquid chromatography tandem mass spectrometry diode array detection
MBC: minimal bactericidal concentration
MIC: minimal inhibitory concentration
MRSA: methicillin‐resistant *Staphylococcus aureus*

MSSA: methicillin susceptible *Staphylococcus aureus*

MTT: 3‐[4,5‐dimethylthiazol‐2‐yl]‐2,5 diphenyl tetrazolium bromide
NIH/3T3: mouse embryonic fibroblasts
OVL/OVl:
*Origanum vulgare* leaves
OVR/OVr:
*Origanum vulgare* rhizomes
QC: quality control
RA: rosmarinic acid
ROS: reactive oxygen species

## 1 Introduction

*Origanum vulgare* L. (family Lamiaceae), known as oregano or wild marjoram, is a woody, perennial, and very tough plant with a straight stem, petiolate and ovate leaves, and white, pink, or light purple flowers. It is native throughout the Mediterranean region, in most parts of the Euro‐Siberian region, and in the Irano‐Turanian region (Benedec et al. 2018; Lukas et al. 2015). Aerial parts of oregano, mainly its leaves, are widely used as a culinary herb or spice (Vallverdú‐Queralt et al. 2014) due to the presence of essential oil, which determines the specific aroma and flavor of the herb (Fleisher and Sneer 1982). Its content is often variable depending on the climatic conditions of plant growth. Thymol, carvacrol, *γ*‐terpinene, *p*‐cymene, *β*‐caryophyllene, limonene, ocimene, linalool, and 4‐terpinenol are its prevalent constituents (Lukas et al. 2015; Mezzomo et al. 2025). However, in addition to essential oil, oregano is a rich source of polyphenols, especially flavonoids (e.g., luteolin, apigenin, and quercetin derivatives) and phenolic acids (particularly rosmarinic acid) (Gutiérrez‐Grijalva et al. 2018). Despite the well‐characterized composition of the aerial parts, the chemical profile of oregano rhizomes has not been investigated so far, making this study the first to explore their phytochemical composition and biological potential.

Oregano and its extracts exhibit a wide range of biological properties, including anti‐inflammatory, antioxidant, antimicrobial, and antiviral effects (Zhang et al. 2014). In self‐medication, oregano is mainly used to alleviate digestive problems, cold, cough, urinary tract diseases, or dental caries (Benedec et al. 2018). In the food industry, oregano as a spice contributes to many factors of food processing like improving the taste, increasing the nutritional value, and improving antioxidant capacity, and due to the content of antibacterial agents, it can consequently reduce microbial growth in food (Vlaic et al. 2022).

Oregano essential oil has been widely documented for its broad‐spectrum antibacterial efficacy against both Gram‐positive and Gram‐negative bacteria, primarily attributed to carvacrol and thymol (György et al. 2020; Saffarian et al. 2024; Stefanakis et al. 2013). However, the majority of these investigations have focused on the volatile oil fraction and leaf extracts, while the underground parts of the plant remain largely unexplored. To date, only limited data are available on the phytochemical profile and biological potential of oregano rhizomes, despite their substantial biomass remaining in the soil after harvesting the aerial parts. This represents not only a loss of potentially valuable bioactive material but also an unexploited opportunity to enhance the overall yield and resource efficiency of oregano cultivation. Given this background, oregano research is highly valuable as the global consumption of oregano is growing annually, with increasing forecasts (MMR 2024).

The exploration of oregano rhizomes as a possible source of phenolic compounds may thus provide new insights into the plant's bioactive potential. Polyphenolic constituents are known to act as natural antioxidants owing to their reducing power, free radical scavenging, and metal‐chelating abilities. In oregano, a strong correlation has been demonstrated between antioxidant capacity and total phenolic content (Gonçalves et al. 2017). Among these, rosmarinic acid exhibits particularly high antioxidant potency, surpassing that of caffeic acid (Baranauskaite et al. 2017). Furthermore, several flavonoids such as vitexin, hyperoside, eriodictyol, (+)‐catechin, and (−)‐epicatechin have been reported as effective radical scavengers (Gonçalves et al. 2017; Radušienė et al. 2010). Besides phenolics, oregano also contains pentacyclic triterpenoids, notably ursolic and oleanolic acids, which contribute to its overall antioxidant activity (Baranauskaite et al. 2016). Considering these findings, comparing the phenolic composition and associated antioxidant effects between rhizomes and leaves could clarify whether the underground organs may serve as an alternative or complementary raw material for extract preparation.

Since biological extracts intended for potential cosmetic, nutraceutical, or pharmaceutical applications must be both active and biocompatible, assessing their cytotoxicity is an essential step. HaCaT human keratinocytes, representing a non‐cancerous epidermal model, were selected to evaluate the safety and compatibility of the rhizome and leaf water extracts with human skin cells (Wiegand and Hipler 2009). This model is particularly relevant for potential topical or dermal formulations containing oregano‐derived ingredients, as it reflects homologous human epidermal tissue for cytotoxicity testing.

Lyophilization, or freeze‐drying, is a dehydration process in which a frozen product is dried by sublimation, removing water without passing through the liquid phase. This prevents most deterioration and microbiological reactions, resulting in a final product of superior quality compared to traditional hot air drying (Ratti 2001). In addition, lyophilization better preserves phenolic compounds and maintains antioxidant capacity compared to standard drying methods, as observed, for example, in spearmint, where freeze‐dried samples retained substantially higher phenolic content and antioxidant activity (Mnerie and Mnerie 2014). This approach allows for more accurate comparison of bioactivity parameters between different plant parts, ensures consistent sample composition across experiments, and provides better control over concentration‐dependent biological effects, which is crucial for reliable assessment of antioxidant and antimicrobial activities.

Taken together, these considerations underline the need for a comprehensive comparative study of oregano rhizomes and leaves. Despite the extensive research on the aerial parts of *Origanum vulgare* , the rhizomes have been largely overlooked, and to our knowledge, no studies have systematically characterized their chemical composition or biological properties. Therefore, this work addresses a significant knowledge gap by providing the first comparative evaluation of *Origanum vulgare* rhizomes and leaves. Specifically, the present study aims to (i) characterize and compare the phenolic composition of both plant parts, (ii) evaluate and contrast their antioxidant and antimicrobial activities, and (iii) assess the cytotoxicity of the extracts on HaCaT cells to determine their safety and potential for biological applications.

## 2 Materials and Methods

### 2.1 Plant Material

The plant ( *Origanum vulgare* L.) was cultivated by the Gene Bank of the Slovak Republic, National Agricultural and Food Centre in Piešťany, Slovak Republic. The aerial parts of five‐year‐old plants were collected at the flowering stage in July 2022, during sunny weather. The rhizomes were dug out of the ground in October 2022. The plants were dried in the shade at room temperature (25°C). The leaves were separated from the stems and flowers. Rhizomes were ground short before extract preparation. The plant material was identified by Ing. Iveta Čičová, PhD., the curator of the Gene Bank of the Slovak Republic. The vouchers (Vouchers Nos OVL_07/2022_GB and OVR_10/2022_GB) are deposited at the Department of Pharmacognosy and Botany, Comenius University Bratislava.

### 2.2 Extract Preparation

The extracts were prepared according to the Czech Slovak Pharmacopeia, 4th edition (PhBs 1987). The final lyophilized (LYO) yields of extracts were 8.46% for OVR and 14.77% for OVL. A detailed description of the method can be found in the Supporting Information.

### 2.3 LC–MS/MS‐Dad

The LC–MS/MS‐DAD analyses of oregano water extracts of leaves or rhizomes were performed on an Agilent 1260 Infinity LC System (Agilent Technologies, Santa Clara, CA, USA), equipped with a binary pump, an autosampler, a column thermostat, and a diode array detector (DAD), coupled to a quadrupole–time‐of‐flight (6520 Accurate‐Mass QTOF) instrument equipped with an orthogonal electrospray ionization source (ESI) (Agilent Technologies, Santa Clara, CA, USA). The separation was performed on a Kromasil C18 column (150 mm × 4.6 mm, 5 μm, Sigma‐Aldrich, Munich, Germany) at 35°C and a flow rate of 0.4 mL/min. Apart from a slight modification, the method was performed, according to (Kubatka et al. 2017). A detailed description of the method can be found in the Supporting Information.

### 2.4 DPPH Radical Scavenging Assay

The antioxidant activities of oregano rhizomes or leaves LYO extracts were estimated spectrophotometrically using a 2,2‐diphenyl‐1‐picrylhydrazyl radical (DPPH^•^) at 515 nm (Blois 1958). A detailed description of the method can be found in the Supporting Information.

### 2.5 ABTS Radical Scavenging Assay

The free radical scavenging activities of oregano rhizomes or leaves LYO extracts were determined spectrophotometrically using the c radical cation (ABTS^•+^) decolorization assay at 515 nm (Re et al. 1999). A detailed description of the method can be found in the Supporting Information.

### 2.6 H_2_O_2_ Scavenging Assay

The H_2_O_2_ scavenging activities of oregano rhizomes or leaves LYO extracts were estimated spectrophotometrically by determination of tris‐1,10‐phenanthroline‐iron (II) complex at 510 nm (Mukhopadhyay et al. 2016). A detailed description of the method can be found in the Supporting Information.

### 2.7 Detection of Intracellular Oxidative Stress

The detection of intracellular oxidative stress was determined according to (Miranda‐Rottmann et al. 2002) with some modifications, using mouse embryonic fibroblasts (NIH‐3T3 cells). A detailed description of the method can be found in the Supporting Information.

### 2.8 Cell Viability by MTT Assay

Cell viability and proliferation were assessed by MTT assay (Šušaníková et al. 2019) using human epidermal keratinocytes (HaCaT cells). A detailed description of the method can be found in the Supporting Information.

### 2.9 Evaluation of Antimicrobial Activity—Broth Microdilution Method of Susceptibility Testing

Seven bacterial collection strains ( *Staphylococcus aureus* CCM 4223, *Staphylococcus aureus* CCM 4750, *Enterococcus faecalis* CCM 4224, *Pseudomonas aeruginosa* CCM 3955, *Escherichia coli* CCM 3954, *Klebsiella pneumoniae* CCM 4415, and *Proteus mirabilis* CCM 7188) were used in the study (for more detailed characteristics, see Table S1). The strains were purchased from the Czech Collection of Microorganisms (Brno, Czech Republic) and kept frozen at −20°C in small aliquots in a cryoprotective medium (Skim Milk, Oxoid, Basingstoke, Hampshire, UK). They were revived prior to testing on blood agar, and the second passage was used in the study.

Oregano LYO extracts were weighed, dissolved in sterile *Aqua pro injectione* to obtain a concentration of 20 mg/mL, and sterilized by filtration. Oxacillin (Oxacillin sodium salt ≥ 19,000 IU/mg, Sigma‐Aldrich, Saint‐Louis, USA) and colistin (Colistin sodium salt > 95%, Sigma‐Aldrich, Saint‐Louis, USA) were used as positive controls. Antibacterial activities were detected according to the EUCAST recommendations (EUCAST 2019) and expressed as the values of the minimal inhibitory concentration (MIC) and minimal bactericidal concentration (MBC).

### 2.10 Microcalorimetry Method of Anti‐Staphylococcal Activity Testing

Biocalorimetry testing of the oregano LYO extracts was performed on the calorimetric instrument calScreener (Symcel, Sweden) using the developed method according to (Beilharz et al. 2023). For this method, we used two bacterial strains of *Staphylococcus*: MSSA and MRSA, listed in Table S1. A detailed description of the method can be found in the Supporting Information.

All tests were performed in three independent runs to exclude the possible excessive values due to permissible measurement error.

The chemicals and all methods used are detailed in the Supporting Information.

### 2.11 Statistical Analysis

The experimental results were statistically analyzed using GraphPad Prism version 5.00 for Windows, GraphPad Software, San Diego, California, USA. Data were expressed as mean ± standard deviation (SD) of triplicate/quadruplicate measurements and were analyzed by Pearson's two‐tailed correlation test. *p* ≤ 0.05 was considered significant. Statistical changes in oregano parts were analyzed by paired, two‐tailed Student's *t*‐test or by ANOVA with Bonferroni post hoc test. *p* ≤ 0.05 was considered significant.

## 3 Results

Extracts of oregano leaves or rhizomes were prepared as an infusion as described above, the most common way of herbal drug preparation in both traditional and conventional use. Water extraction of oregano parts yields different extract concentrations, specifically 8.46 and 14.77 mg/mL for oregano rhizomes and leaves, respectively.

### 3.1 Phytochemical Analysis of Extracts

The phenolic compounds present in the samples were characterized according to their UV and mass spectra displayed in Table 1. Caffeic acid (1), luteolin‐7‐*O*‐diglucuronide (2), lithospermic acid (4), rosmarinic acid (10), salvianolic acid A (11), and salvianolic acid B (12) were identified by comparing their retention time, mass, and UV–VIS spectra with commercial standards and available literature. Respective chromatograms of LC–MS/MS‐DAD are presented in Figure S1.

**TABLE 1:** Polar phenolic compounds in oregano extract, their corresponding retention times (TR), molecular ions [M‐H], and MS^2^ fragments in LC–MS/MS analysis and quantitative abundance of polar phenolic compounds (mg.g^−1^).

Peak	Componud	T_R_ (min)	[M‐H]^ˉ^ (*m/z*)	MS^2^ (20 eV) (*m/z*)	UV_max_ (nm)	Mass concentration (mg.g^−1^)^a^ ± SD	
						*O. vulgare* leaves	*O. vulgare* rhizomes
1	Caffeic acid	8.553	179.0355	135.0456	325, 290	LOQ	LOD
2	Luteolin‐7‐*O*‐diglucuronide	12.487	637.1037	351.0439 285.0404 175.0327	265, 350	2.79 ± 0.04	ND
3	Salvianolic acid K	13.14	555.1171	537.1023 493.1155 295.0599 227.0629 185.0250 161.0201	277, 325	ND	LOQ
4	Lithospermic acid	13.333	537.1028	295.0663 179.0280 135.087	250, 280, 340	LOQ	ND
5	Rosmarinic acid hexoside	14.553	521.1303	359.1231 197.0410 161.0214	237, 285, 325	LOQ	ND
6	4´‐*O*‐β‐D‐diglucosyl‐4′‐hydroxybenzyl protocatechuate	15.453	583.1667	421.1131 153.0197	260	ND	1.34 ± 0.01
7	Rosmarinic acid hexoside	15.9	521.1295	359.0763 323.0770 197.0451 161.0232	237, 283, 325	LOQ	ND
8	4´‐*O*‐β‐D‐glucopyranosyl‐3′0.4′‐dihydroxybenzyl protocatechuate (oreganol A)	17.027	437.1093	153.0191	265, 300	8.22 ± 0.08	ND
9	4´‐*O*‐β‐D‐glucopyranosyl‐4′‐hydroxybenzyl protocatechuate (oreganol C)	17.360	421.1150	153.0198	260	3.39 ± 0.12	1.70 ± 0.04
10	Rosmarinic acid	18.127	359.078	197.0455 179.0338 161.0250 135.0460	330, 290, 237	20.07 ± 0.24	7.07 ± 0.01
11	Salvianolic acid A	19.06	493.1161	295.0615 313.0683 185.0249 135.0459	310, 287, 254, 228	ND	18.29 ± 0.10
12	Salvianolic acid B	20.86	717.1446	519.0949 321.0366 185.022	320, 286, 240	ND	LOQ
13	4´‐*O*‐β‐D‐glucopyranosyl‐3′0.4′‐dihydroxybenzyl 4‐*O*‐methylprotocatechuate (oreganol B)	21.780	451.1246	167.0343 152.0110	265, 285, 300	4.78 ± 0.13	ND
14	Salvianolic acid A isomer	21.979	493.1163	313.0972 295.0575 197.0497 185.0229 159.0465	310, 287, 254, 228	ND	LOQ

Abbreviations: LOD, limit of detection; LOQ, limit of quantification; ND, not detected.

^a^ Values (mg/g dry extract − lyophilizate) are presented as means ± standard deviation (*n* = 3); used external standards were: luteolin (used for flavonoids determination, *λ* = 360 nm) and rosmarinic acid (used for the determination of phenolic acids and their derivatives, *λ* = 280 nm or *λ* = 320 nm).

The quantification of identified compounds was performed using external standards (luteolin and rosmarinic acid). The proposed method was validated with the sensitivity and precision parameters. All standards showed good linearity. The following *r* ^2^ values were obtained: rosmarinic acid at 280 nm *r* ^2^ = 0.9998, regression curve *y* = 259.44*x* − 226.01, LOD = 0.53 μg/mL and LOQ = 1.77 μg/mL; rosmarinic acid at 320 nm *r* ^2^ = 0.9986, regression curve *y* = 391.7*x* + 168.23, LOD = 1.48 μg/mL and LOQ = 4.94 μg/mL; luteolin *r* ^2^ = 0.9997, regression curve *y* = 359.49*x* + 310.88, LOD = 0.01 μg/mL and LOQ = 0.04 μg/mL.

Six compounds were authenticated using commercial standards. [M‐H]ˉ ion 555.1171 in 13.14 min (peak 3) with a major fragment at 295.0599 indicated the presence of salvianolic acid K (Taghouti et al. 2020) in underground parts of oregano but not detected in leaves. Peak 5, which was detected in leaves, generated a pseudomolecular ion [M‐H]ˉ ion at m/z 521.1303 with a fragment at m/z 359.1231 (loss of hexosyl moiety 162 Da) and fragments at m/z 197.0451 (M‐H‐caffeoyl‐glucosyl) and 161.0214 (M‐H‐dihydroxyphenyllactoyl‐glucosyl). This peak was identified as rosmarinic acid hexoside (Budzianowski et al. 2023). Similar spectra were obtained for the same sample in 15.9 min; we presume rosmarinic acid hexoside with different sugar positions. Similar spectra but different retention times have shown peaks 11 and 14. Compound 14, identified in oregano rhizomes, is supposed to be a salvianolic acid A isomer (Taghouti et al. 2020). Peaks 6, 8, 9, and 13 were identified as protocatechuic acid esters: 4′‐*O*‐β‐D‐diglucopyranosyl‐4′‐hydroxybenzyl protocatechuate with [M‐H]ˉ ion at *m*/*z* 583.1667 and fragment ion 421.1131 (M‐H‐glucosyl); 4′‐*O*‐ß‐D‐glucopyranosyl‐3′0.4′‐dihydroxybenzyl protocatechuate (oreganol A) with [M‐H]ˉ ion at *m*/*z* 437.1093; 4′‐*O*‐β‐D‐glucopyranosyl‐4′‐hydroxybenzyl protocatechuate (oreganol C) with pseudomolecular ion [M‐H]ˉ at *m*/*z* 421.1150 and 4′‐*O*‐β‐D‐glucopyranosyl‐3′0.4′‐dihydroxybenzyl 4‐*O*‐methylprotocatechuate (oreganol B) with pseudomolecular ion [M‐H]ˉ at *m*/*z* 451.1246, respectively. The presence of protocatechuic acid derivatives was described in the genus *Origanum* in the past (Kubatka et al. 2017; Matsuura et al. 2003) and seems to be typical for oregano. Notably, three compounds, caffeic acid, oreganol C, and rosmarinic acid, were present in both plant parts. In the quantitative analysis, the predominant compounds in the leaves (OVL) were rosmarinic acid, oreganol A, oreganol B, oreganol C, and luteolin‐7‐*O*‐diglucuronide. Salvianolic acid A, rosmarinic acid, and oreganol C were the major compounds found in the rhizomes (OVR).

### 3.2 In Vitro Antioxidant Capacity

In vitro antioxidant capacities of oregano rhizomes or leaves LYO extracts were estimated spectrophotometrically using three different scavenging assays. In ABTS and DPPH assays, the mechanism of action is established on hydrogen atom transfer and single electron transfer between antioxidant and radical (Kurin et al. 2012; Rumpf et al. 2023). In the third assay, we determined the ability of both LYO extracts to scavenge H_2_O_2_ in the presence of Fe (II) ion (Mukhopadhyay et al. 2016).

**FIGURE 1:**
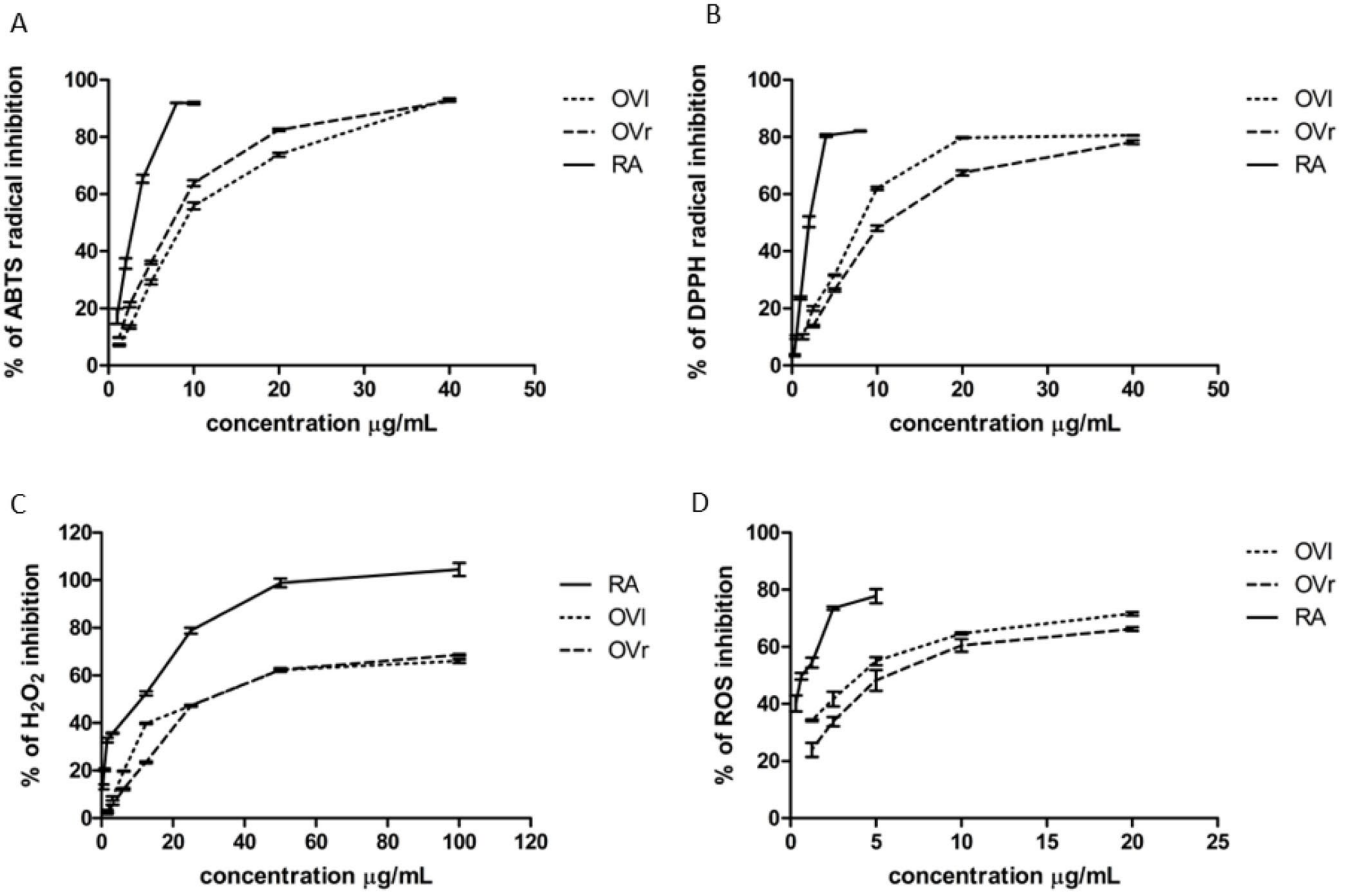
(A) Dose‐dependent ABTS^•+^ inhibition of *Origanum vulgare* leaves (OVL) or rhizomes (OVR) LYO extracts or rosmarinic acid (RA). (B) Dose‐dependent DPPH^•^ inhibition of *O. vulgare* leaves (OVL) or rhizomes (OVR) LYO extracts or rosmarinic acid (RA). (C) Dose‐dependent H_2_O_2_ inhibition of *O. vulgare* leaves (OVL) or rhizomes (OVR) LYO extracts or rosmarinic acid (RA). (D) Dose‐dependent DCF production inhibition of *O. vulgare* leaves (OVL) or rhizomes (OVR) LYO extracts or rosmarinic acid (RA). The bars represent mean ± SD, *n* = 4; *p* < 0.05 (Pearson's two‐tail correlation test).

As shown in Figure 1A–C, respectively, both LYO extracts exhibited dose‐dependent antioxidant activity. In ABTS assay was IC_50_ (concentration needed for 50% inhibition of radical) of OVL 11.86 μg/mL (*r* ^2^ = 0.99) and of OVR 7.55 μg/mL (*r* ^2^ = 0.99), respectively. In this assay, oregano rhizomes LYO extract was significantly more active than the LYO extract of leaves (*p* ≤ 0.05, Student's *t*‐test). In the DPPH model was IC_50_ of OVL 6.32 μg/mL (*r* ^2^ = 0.97) and of OVR 8.50 μg/mL (*r* ^2^ = 0.99). In this case, oregano leaves were reversely significantly more active than oregano rhizomes (*p* ≤ 0.05, Student's *t*‐test). In the H_2_O_2_ scavenging model, the IC_50_ of OVL was 10.08 μg/mL (*r* ^2^ = 0.99) and of OVR 22.66 μg/mL (*r* ^2^ = 0.99). Remarkably, we observed a very early onset of the plateau already at the level of approximately 65% of the effect, whereas rosmarinic alone reached 100% at the concentration slightly above 50 μg/mL. The antioxidant activity of rosmarinic acid, which was used as a positive control, was stronger compared to LYO extracts in all assays. IC_50_ of rosmarinic acid in ABTS assay was 3.27 μg/mL e q. 9.08 μM (*r* ^2^ = 0.99), in DPPH one 1.83 μg/mL e q. 5.08 μM (*r* ^2^ = 0.97), and in H_2_O_2_ scavenging model was 19.40 μg/mL e q. 53.84 μM (*r* ^2^ = 0.98), respectively.

### 3.3 Detection of Intracellular Oxidative Stress

The amount of formed intracellular ROS (reactive oxygen species) is reflected by the intensity of fluorescence of DCF (2′,7′‐dichlorofluorescein) (Hadrich et al. 2016). In our experiments, H_2_O_2_ was used as the activator of intracellular oxidative stress. After 15 min of incubation, it caused a 2‐fold increase in ROS level compared to the control cells (data not shown), which reflects previous studies (Bittner Fialová et al. 2020; Miranda‐Rottmann et al. 2002; Trajčíková et al. 2020). Both tested LYO extracts and rosmarinic acid (used as a positive control) significantly reduced the ROS production of NIH/3T3 cells (mouse embryonic fibroblasts) treated with H_2_O_2_ after 1 h of incubation when compared to the control. As can be seen in Figure 1D, both oregano LYO extracts exhibited dose‐dependent antioxidant activity. IC_50_ of OVL was 3.95 μg/mL (*r* ^2^ = 0.99) and one of was OVR 3.23 μg/mL (*r* ^2^ = 0.98), the IC_50_ of rosmarinic acid was 1.57 μg/mL e q. 4.36 μM (*r* ^2^ = 0.95). The LYO extract of leaves was significantly more active than that of rhizomes (*p* ≤ 0.05, Student's *t*‐test). Similarly to the H_2_O_2_ scavenging assay, we observed an early onset of the plateau already at the level of approximately 60% of the effect, whereas rosmarinic alone was able to reach 80%.

### 3.4 Cell Viability by MTT Assay

**FIGURE 2:**
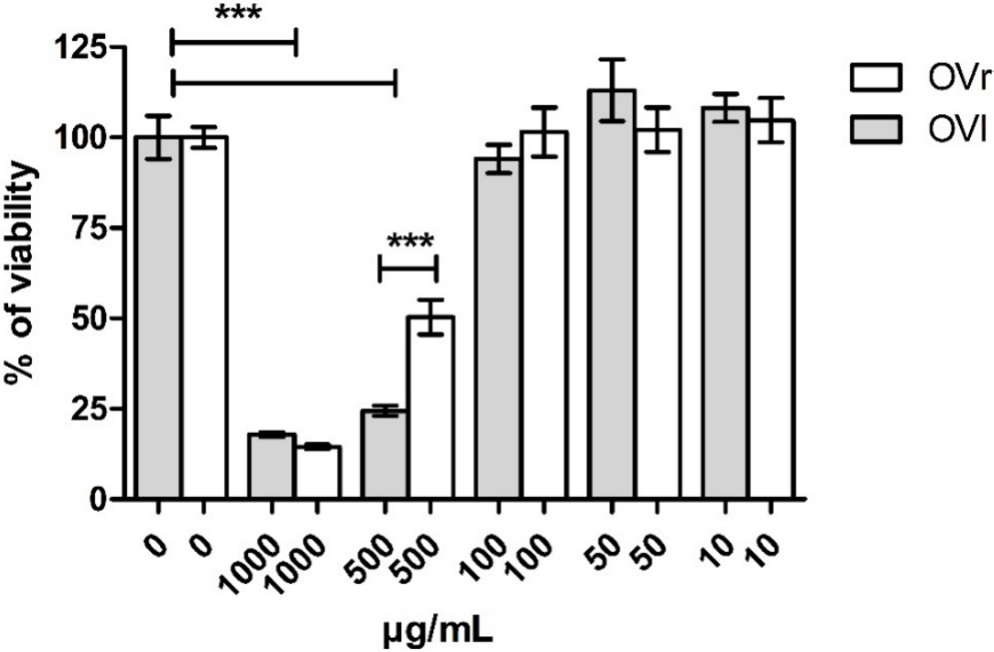
Percentage of cell viability after 72 h *Origanum vulgare* leaves (OVL) or rhizomes (OVR) LYO extracts treatment on HaCat cells assessed by MTT test. The bars represent mean ± SD, *n* = 3; ***p < 0.001 against untreated control (ANOVA/Bonferroni). Untreated cells (0 μg/mL) served as the negative control.

MTT colorimetric assay was used to evaluate the effect of OVL and OVR on cell viability. HaCaT cells (human epidermal keratinocytes) (Boukamp et al. 1988) were treated for 72 h with both extracts in different concentrations (10, 50, 100, 500, 1000 μg/mL). As can be seen in Figure 2 both oregano extracts reduce cell viability in a dose‐dependent manner. However, they did not significantly decrease cell viability at a concentration ≤ 100 μg/mL compared to untreated cells. At the concentration of 500 μg/mL, cell viability was significantly higher with OVR than with OVL treatment. The calculated IC_50_ value of irinotecan hydrochloride (positive control), an anti‐proliferative agent, was 45.38 μM (Figure S3).

### 3.5 Evaluation of Antimicrobial Activity

Antimicrobial activity testing was performed using the broth microdilution method and expressed as minimal inhibitory and minimal bactericidal (MIC and MBC, respectively). The results are shown in Table 2. The highest antimicrobial activity was observed against *S. aureus* strains, where MICs and MBCs of both the OVL and OVR were 2.5 mg/mL. Of the Gram‐negative bacteria, *P. mirabilis* was the most susceptible, where the OVL MIC and MBC were 2.5 mg/mL (similar to *S. aureus* strains), whereas the MIC and MBC of OVR were twice as high (5 mg/mL). The tested LYO extracts also inhibited the growth of *E. faecalis* (MIC 10 mg/mL for OVL, MIC 2.5 mg/mL for OVR), but they were not bactericidal against *E. faecalis* at the tested concentration range (MBC > 10 mg/mL). *P. aeruginosa* , *E. coli* , and *K. pneumoniae* were found to be resistant to both LYO extracts in the tested concentration range.

**TABLE 2:** MICs and MBCs of tested oregano LYO extracts in mg/mL and oxacillin and colistin as positive controls.

Bacterial species	OVL (mg/mL)	OVR (mg/mL)	Oxacillin (mg × 10^−4^/mL)	Colistin (mg × 10^−4^/mL)
	MIC/MBC	MIC/MBC	MIC/MBC	MIC/MBC
*Staphylococcus aureus* (MSSA)	2.5/2.5	2.5/2.5	1.25/2.5	1250/1250
*Staphylococcus aureus* (MRSA)	2.5/2.5	2.5/2.5	5/20	1250/2500
*Enterococcus faecalis*	10/> 10	2.5/> 10	125/250	2500/> 2500
*Pseudomonas aeruginosa*	> 10/> 10	> 10/> 10	nd	3/10
*Escherichia coli*	> 10/> 10	> 10/> 10	nd	3/10
*Klebsiella pneumoniae*	> 10/> 10	> 10/> 10	nd	nd
*Proteus mirabilis*	2.5/2.5	5/5	nd	2500/> 2500

Abbreviations: MBC, minimal bactericidal concentration; MIC, minimal inhibitory concentration; nd, not detected.

**FIGURE 3:**
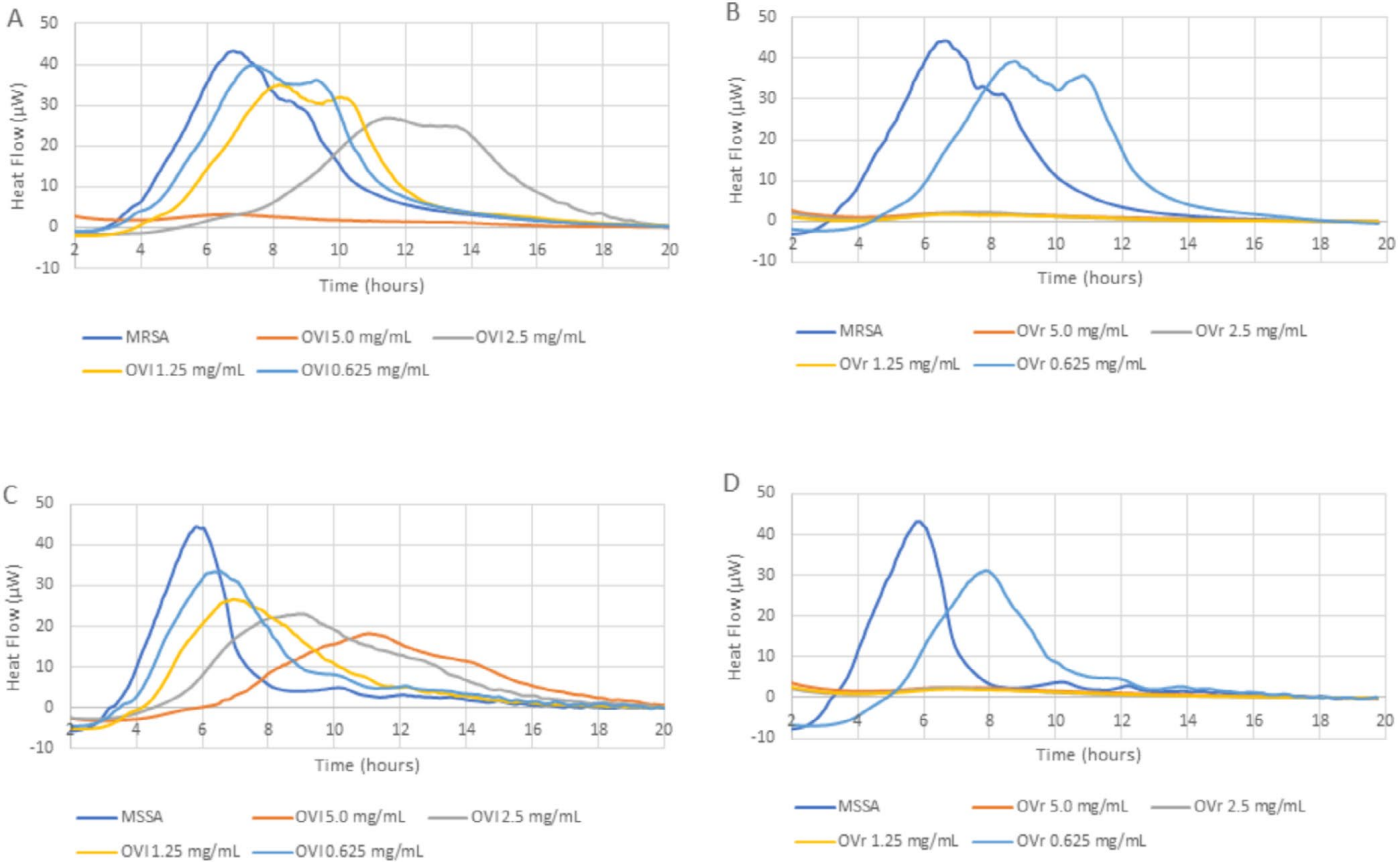
Heat flow diagrams displaying the metabolic activity of MRSA in BHI broth without treatment and impacted by OVL (A) or OVR (B) and the metabolic activity of MSSA in BHI broth without treatment and impacted by OVL (C) or OVR (D). All extracts were in the 0.625–5.0 mg/mL concentration range.

**FIGURE 4:**
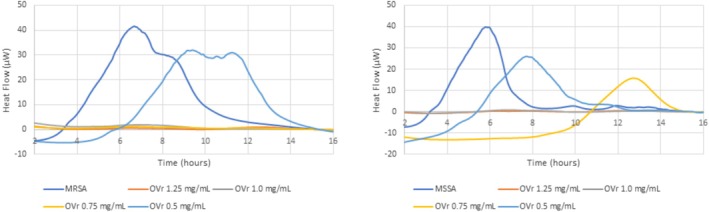
Heat flow diagrams displaying MRSA (left) and MSSA (right) metabolic activity in BHI broth without treatment and impacted by OVR in the concentration range of 0.5–1.25 mg/mL.

**FIGURE 5:**
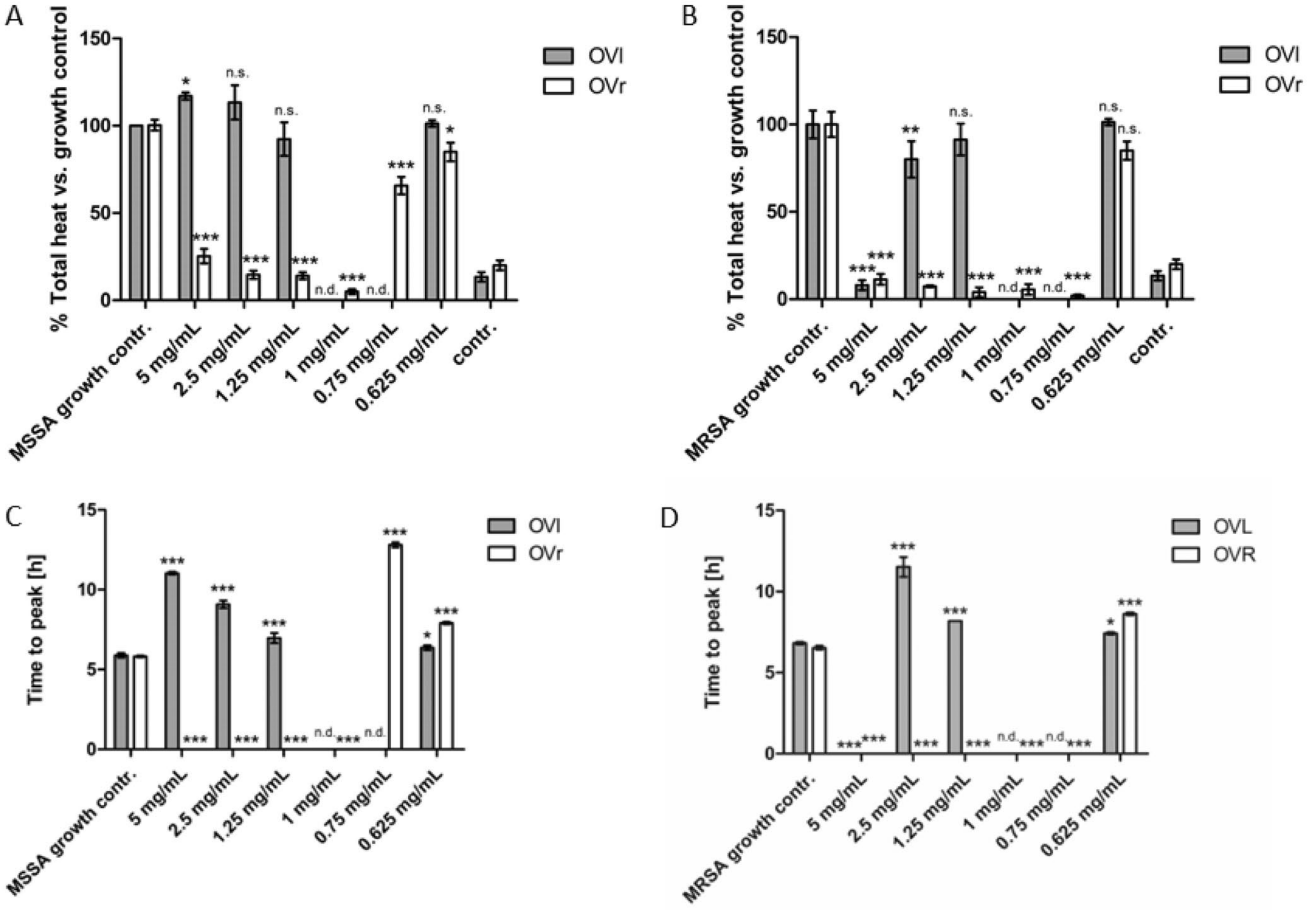
Total Heat vs. growth control MSSA (A), MRSA (B), and Time to Peak MSSA (C) and MRSA (D) values acquired by IMC, reflecting anti‐staphylococcal effect of OVL and OVR. The bars represent mean ± SD, *n* = 3; ***p ≤ 0.001, **p ≤ 0.01, *p ≤ 0.05, n.s. = *p* > 0.05 against untreated control (ANOVA/Bonferroni). Not determined samples are expressed as n.d.

Based on the results from broth microdilution method testing, the antibacterial activity of OVL and OVR using microcalorimetry was tested on two “most susceptible” bacterial strains: MSSA and MRSA. The antibacterial activity is expressed as Heat Flow in Figures 3A–D and 4 and by parameters of Total heat (Figure 5A,B) and Time to peak (Figure 5C,D). Based on the results of the broth microdilution assay, oxacillin was used as a positive control in the microcalorimetry assay at concentrations of 1 μg/mL for MRSA and 0.25 μg/mL for MSSA. Heat flow thermograms for these control conditions are shown in Figure S2. As the Heat Flow values for oxacillin‐treated samples remained below 10 μW and did not exceed the 20% threshold for relative heat ratio (Tellapragada et al. 2020) the corresponding Total Heat and Time to Peak values were negligible and therefore not considered for further quantitative comparison.

As for the used concentrations (serially diluted) of OVR, the “break” of antibacterial action was unclear, so additional dilution was performed, and the results are displayed in Figure 4.

Considering all parameters (Heat Flow, Total Heat, Time to Peak) in Figures 3, 4, 5, the metabolic activity of MRSA and MSSA was inhibited by oregano leaves in concentration ≥ 5.0 mg/mL, while the oregano rhizomes inhibited the metabolic rate of staphylococci strains in six‐fold lower concentrations ≥ 0.75 mg/mL (Figure 4).

## 4 Discussion

Qualitative and quantitative analyses were conducted utilizing LC–MS/MS‐DAD. The qualitative analysis identified 14 phenolic compounds (Figure S1), with nine detected in the leaves and eight in the rhizomes (Table 1). The phytochemical composition of the aerial parts of *Origanum vulgare* is well documented, having been extensively characterized in earlier studies (Kubatka et al. 2017; Martins et al. 2014). Available literature is fully consistent with our findings, confirming the presence and relative abundance of secondary metabolites in water, hydroalcoholic, and alcoholic extracts of the aerial parts of *O. vulgare* , where rosmarinic acid and other cinnamic acid derivatives are accompanied by flavonoids mainly represented by luteolin, quercetin, kaempferol, and apigenin derivatives (Martins et al. 2014; Wahab et al. 2025). In contrast, the underground parts of *O. vulgare* remain insufficiently explored with respect to both their phytochemical profile and biological activity. This work was specifically conceived to fill this evident knowledge gap by providing the first systematic experimental evaluation of the phytochemical composition and biological activity of *O. vulgare* underground parts. Our results clearly demonstrate that these plant underground organs also constitute a rich source of bioactive secondary metabolites, with phenolic acids being the dominant class.

Oxidative stress is one of the factors in the development of many aging‐related diseases, such as cancer, neurodegenerative diseases, and cardiovascular diseases. Antioxidants are compounds capable of reducing or neutralizing oxidative stress, and therefore, they are studied for therapeutic purposes (de Torre et al. 2020; Gracia et al. 2017; Kim et al. 2015; Saha et al. 2017). However, oxidation is undesirable in the food industry as well because it can shorten the shelf life of stable foods, including ingredients, raw materials, and semi‐manufactured products, and make them less acceptable or totally unacceptable to consumers. Moreover, oxidative reactions can cause the formation of toxic compounds in some cases (Calligaris et al. 2016). Therefore, the search for antioxidant substances of plant origin makes sense both for the pharmaceutical and food industries, especially if they can be obtained ecologically from waste, such as, for example, otherwise unused underground parts of plants. The antioxidant activity of *Origanum vulgare* leaves, whether in ethanol extracts or essential oil form, has been extensively studied (Baranauskaite et al. 2017; Coccimiglio et al. 2016; Han et al. 2017; Moghrovyan et al. 2019; Quiroga et al. 2011; Radušienė et al. 2010). Similarly, the antioxidant properties of essential oil derived from *Origanum vulgare* rhizomes have also been explored (Han et al. 2017; Morshedloo et al. 2018). Additionally, research has compared the antioxidant activity of water extracts from *O. vulgare* leaves to that of ethanolic and methanolic extracts (Cervato et al. 2000; Chun et al. 2005; Koldaş et al. 2015). However, to the best of our knowledge, no previous studies have examined the biological activities of water extracts (in lyophilized form) obtained from *Origanum vulgare* rhizomes. Although we did not measure the antioxidant activity of the compounds that were identified in the extracts (caffeic acid, luteolin‐7‐*O*‐diglucuronide, salvianolic acid A, oreganol A, oregano C), they all share the characteristic presence of the catechol group with hydroxyl groups (OH) in the 3,4‐ortho position, which is responsible for antioxidant activity (De La Cruz et al. 2015). This suggests that, despite the well‐known antioxidant activity of the essential oil (Quiroga et al. 2011; Teixeira et al. 2013), the secondary metabolites in water extracts of oregano, both from the leaves and rhizomes, are significant carriers of antioxidant effects and contribute to the plant's overall activity. We identified rosmarinic acid in both the leaves and rhizomes of oregano and utilized it as a positive control in DPPH and ABTS antioxidant assays with strong measured activity. In addition, we observed that rosmarinic acid prevented the oxidation of Fe(II) to Fe(III) in the presence of hydrogen peroxide, further confirming the plateau at the 100% level. Therefore, we assume that this effect arises from either a direct interaction of rosmarinic acid with H_2_O_2_, protecting Fe(II) from oxidation, or from the coordination of Fe(III) by polyphenols, which may lead to the reduction of Fe(III) and the formation of semiquinones, ultimately producing quinone species and Fe(II) (Ndomou and Mube 2023) which can subsequently bind to 1,10‐phenanthroline. In both oregano extracts, we observed a lowered plateau in the H_2_O_2_ scavenging assay. Oregano LYO extracts are a broad mixture of secondary metabolites. Phenolics, which contain catechol and galloyl fragments in their structure, can form stable coordination‐saturated complexes with metals of variable valency and then do not react with the H_2_O_2_ to form the hydroxyl radical (Ivanova et al. 2020). In the presence of dissolved oxygen in solution, Fe(II)‐polyphenol complexes are rapidly oxidized to give Fe(III)‐polyphenol complexes, which cannot participate in the Fenton reaction (Perron et al. 2010). Therefore, part of the iron cannot enter the reaction, which leads to a decrease in the plateau. On the other hand, if the internal redox reaction of the Fe(III)–catechol complex leads to the formation of Fe(II) and quinone in some cases (Sánchez et al. 2005), thus the formation of tris‐(1,10‐phenanthroline)iron(II) complex does not depend entirely on the ability of secondary metabolites to scavenge hydrogen peroxide. In living cells, H_2_O_2_ is made in mitochondria as a reactive byproduct through the electron transport chain. Like other reactive oxygen species, it participates in various processes, including inflammatory responses and cellular senescence (Hahn et al. 2017). Some cell‐based experiments have shown protective effects of oregano essential oil against H_2_O_2_‐induced cell damage (Y. Zou et al. 2016; Cheng et al. 2018). To date, no one has investigated the effect of aqueous extracts of oregano leaf and rhizome on H_2_O_2_‐induced oxidative stress in mouse embryonic fibroblasts (NIH/3T3). We confirmed dose‐dependent protective antioxidant effects of both OVL and OVR, as well as rosmarinic acid, on H_2_O_2_‐induced oxidative injury in cells treated with H_2_O_2_. However, like in vitro H_2_O_2_ scavenging assay, early onset of the plateau at the level of approximately 60% of the total effect was observed in contrast to rosmarinic acid alone.

As we have already mentioned in the results, oregano rhizomes demonstrated similar antioxidant activity in all in vitro measurements. Therefore, rhizomes have the potential to become an antioxidant equivalent to the leaves, with a significant impact on recycling and sustainability.

MTT colorimetric assay was used to evaluate the effect of OVL and OVR on cell viability. HaCaT cells (human epidermal keratinocytes) were treated for 72 h with both extracts in different concentrations (10, 50, 100, 500, 1000 μg/mL). As can be seen in Figure 2 oregano extracts reduce cell viability in a dose‐dependent manner. Neither extract significantly decreased cell viability at a concentration of 100 μg/mL and was lower compared to the negative control (untreated cells). At the concentration of 500 μg/mL, cell viability was significantly higher with OVR than with OVL treatment. These results indicate that both oregano extracts exert only mild cytotoxicity at lower concentrations, suggesting an overall favorable safety profile. Cell viability was not significantly affected up to 100 μg/mL for either extract, while a moderate, dose‐dependent decrease was observed at higher concentrations. Notably, OVR maintained significantly higher cell viability at 500 μg/mL compared to OVL, indicating a comparable or slightly improved biocompatibility. This finding is particularly relevant, as it demonstrates that the previously underexplored underground parts of oregano exhibit not only antioxidant and antimicrobial potential but also a safety profile similar to that of the traditionally used leaves.

The activity of oregano leaves and rhizomes against different bacterial strains was tested using two different methods: conventional broth microdilution and isothermal microcalorimetry (IMC). From the first, we obtained the MIC and MBC parameters, while the second is highly sensitive and detailed monitors the real‐time metabolic process of bacteria that generates heat in microwatts (μW). As Braissant mentioned, heat flow records the metabolic processes (e.g., glucose respiration, glucose fermentation, lactose fermentation, etc.) of bacterial growth—the generation time, which depends on environmental factors such as nutrient availability and environmental temperature (Braissant et al. 2010). Generation time may differ for different bacteria (Liptáková 2023). Bacterial growth, alongside all chemical and physical processes, results in a heat flow signal. The produced heat may be measured and mathematically calculated (Astasov‐Frauenhoffer et al. 2012; Braissant et al. 2010). As for our results in Table 2, the antimicrobial activity of *Origanum vulgare* leaves and rhizomes was very similar. In general, Gram‐positive bacterial strains were more susceptible than Gram‐negative ones. However, while the microdilution method offered the sole value of minimal inhibitory (MIC_MSSA/MRSA_ = 2.5 mg/mL) or minimal bactericidal concentration (MBC_MSSA/MRSA_ = 2.5 mg/mL), IMC provided, in real‐time, quantitative data on all metabolic processes that produce or consume heat. The principle of IMC is the direct and continuous measurement of the metabolic rate of living cells through a real‐time heat flow profile, which allows the mapping of the extract's impact on bacteria in time. IMC generates a continuous real‐time electronic signal proportional to the heat produced by an ampoule of microorganisms, enabling ongoing observation of fluctuations in their metabolic activity and replication rates (Braissant et al. 2010), IMC resulted in heat flow vs. time curves and other parameters (Time to Peak, Total Heat) that showed not only an inhibitory effect but also informed about delays in bacteria metabolic activity (Time to Peak) and overall metabolic rate (Total Heat)—decreased or even increased when treated by different extract concentrations. Heat flow and activity indicate metabolic rates, while total heat also reflects the amount of substrate consumed or metabolic products released (Braissant et al. 2010). A detailed examination of bacterial behavior in relation to metabolic processes and their modulation by extracts not only enhances the understanding of microbial dynamics but also informs the rational design of dosing strategies for medicinal extracts, ultimately improving their therapeutic potential. Surprisingly, the rhizome extracts of oregano demonstrated significantly stronger antibacterial activity against MSSA and MRSA strains in the more sensitive IMC assay, showing a six‐fold higher efficacy compared to the leaf extracts (≥ 0.75 vs. ≥ 5.0 mg/mL, respectively). This differs from the results of the broth microdilution test, where both extracts showed comparable MIC values. Showing discrepancy highlights the higher sensitivity and resolution of IMC, which can detect subtle changes in bacterial metabolic activity that are not always observable with conventional visual‐based assays. Nevertheless, the data consistently indicate that oregano rhizomes possess at least equal, if not superior, anti‐staphylococcal potential compared to the traditionally used leaves. This may be attributed to their distinct phytochemical profile, particularly their high content of phenolic acids, chiefly salvianolic acid A. Given that oregano rhizomes are not currently utilized in the food or pharmaceutical industries, our findings suggest that they represent a valuable and underexplored plant source of bioactive compounds with antibacterial potential. The available literature on the antimicrobial activity of oregano primarily assesses its essential oil, which was tested against a broad spectrum of different strains of *Escherichia coli* , *Saccharomyces cerevisiae* , *Vibrio anguillarum* (Stefanakis et al. 2013), *Klebsiella oxytoca* , *Klebsiella pneumoniae* (Fournomiti et al. 2015), *Acinetobacter baumannii*, *Pseudomonas aeruginosa* , methicillin‐resistant *Staphylococcus aureus* (Lu et al. 2018), *Staphylococcus epidermidis* , *Klebsiella aerogenes* , *Streptococcus mutans* (Verma et al. 2012), *Streptococcus pneumoniae* , *Enterococcus faecalis* (Mohsen et al. 2022). In addition to the essential oil, *Origanum vulgare* is traditionally used for therapeutic purposes in the form of aqueous infusions and decoctions, which have also been shown to exhibit pronounced antioxidant and antibacterial activities. As reported by Martins et al. (2014), the infusion, decoction, and hydroalcoholic extract of *Origanum vulgare* L. demonstrated antibacterial efficacy against *Staphylococcus epidermidis* , *Escherichia coli* , *Pseudomonas aeruginosa* , *Enterobacter sakazakii* , and *Proteus vulgaris* at a concentration of 20 mg/mL.

Rosmarinic acid, the primary compound in oregano leaves, and salvianolic acid A in rhizomes are notable hydroxycinnamic acids with established antibacterial potential. Rosmarinic acid, derived from caffeic and dihydrocaffeic acids, demonstrates bacteriostatic effects by targeting bacterial proteins and inhibiting Na^+^/K^+^‐ATPase activity (Kernou et al. 2023; Wang et al. 2024). Rosmarinic acid can be transformed in plants into salvianolic acids under the specific action of enzymes and other reactions (Wang et al. 2019). Salvianolic acid A was the dominant compound present in oregano aqueous rhizome extracts. It has already been shown that even salvianolic acid A did not affect directly the growth of *Staphylococcus aureus* (MRSA); it has several associated effects that reduce the lethality of infection in mice, like: sortase A inhibition, suppression of bacterial adhesion to fibrinogen and attachment of protein A to the cell wall, inhibition of biofilm formation, and bacterial invasion into human lung cells (Mu et al. 2020). As mentioned, salvianolic acid A was identified as the prevailing compound in the underground parts. This molecule has emerged as a highly promising natural compound that has attracted considerable scientific attention in recent years. A broad spectrum of biological activities has been reported for salvianolic acid A, including β‐lactamase inhibition (Yu et al. 2018), antiviral activity (Hu et al. 2021), cardioprotective effects (Li et al. 2023; Zhou et al. 2020), anti‐inflammatory activity (Zou et al. 2023), and antioxidant properties (Khan et al. 2025). Overall, our findings support the growing body of evidence indicating that salvianolic acids, which are particularly widespread within the family Lamiaceae, represent a highly promising group of natural compounds with significant beneficial biological effects (Yang et al. 2025).

Plant‐derived compounds often exhibit both antimicrobial and antioxidant properties, but these activities are not always directly correlated (Ispiryan et al. 2024). While reactive oxygen species are central to the bactericidal mechanisms of conventional antibiotics (Dwyer et al. 2014), plant‐based antioxidants could counteract these effects. The outcome depends on factors such as the penetration rates of antioxidant molecules and their interaction with bacterial structures. Nonetheless, antioxidants play a protective role for host tissues, mitigating oxidative damage during inflammation associated with microbial infections (Rodríguez‐Yoldi 2021). Our findings also indicate that the full extract is required because the combined constituents seem to act synergistically and amplify the effect compared to individual isolated compounds.

Comprehensive pharmacological profiling and synergy‐antagonism studies with antibiotics are essential to optimize the clinical application of oregano leaves and/or rhizomes extracts. This dual activity, spanning antibacterial and antioxidant effects, positions oregano water extracts as promising candidates for therapeutic use. Limitations of this study include the unclear mechanisms behind the stronger antibacterial activity of rhizome extracts and the narrow range of tested microorganisms. Future research should address these aspects and explore practical applications of oregano rhizome extracts in food and medical fields.

## 5 Conclusion

This study provides a detailed assessment of the antioxidant and antibacterial properties of water extracts derived from *Origanum vulgare* leaves and rhizomes, using a diverse set of analytical methodologies. The findings indicate that antioxidant activity was comparable between leaf and rhizome extracts across both chemical and cellular assays. However, the efficacy of these extracts was determined to be approximately 2–4 times lower than that of rosmarinic acid, depending on the experimental model utilized. In antibacterial assessments, the conventional broth microdilution assay did not reveal significant differences in anti‐staphylococcal activity between leaf and rhizome extracts. In contrast, the more sensitive intracellular concentration monitoring assay demonstrated a markedly stronger antibacterial effect for rhizome extracts, suggesting a potential advantage in antimicrobial applications. Importantly, cytotoxicity testing on HaCaT cells confirmed that rhizome extracts exhibit a safety profile comparable to that of leaf extracts, indicating good biocompatibility and supporting their potential use in formulations intended for human applications. These results highlight *O. vulgare* rhizomes as an underexplored yet valuable source of hydroxycinnamic derivatives, particularly salvianolic acid A. Their water extracts demonstrate antioxidant, antimicrobial, and biocompatibility profiles comparable to, or in some aspects exceeding, those of the aerial parts. Considering the growing interest in resource efficiency and the utilization of plant materials beyond conventionally harvested organs, oregano rhizomes represent a promising candidate for further investigation in pharmaceutical and biomedical research.

## Author Contributions

**Elena Kurin:** conceptualization (equal), data curation (equal), formal analysis (equal), investigation (equal), methodology (equal), validation (equal), visualization (equal), writing – original draft (equal), writing – review and editing (equal). **Kamila Dokupilová:** data curation (equal), investigation (equal). **Ema Kostovčíková:** data curation (equal), investigation (equal). **Lívia Slobodníková:** data curation (equal), investigation (equal), writing – original draft (equal). **Eva Drobná:** data curation (equal), investigation (equal). **Iveta Čičová:** investigation (equal). **Veronika Brindza Lachová:** data curation (equal), investigation (equal). **Jana Sabová:** investigation (equal). **Peter Gál:** investigation (equal). **Milan Nagy:** supervision (equal), visualization (equal), writing – review and editing (equal). **Pavel Mučaji:** funding acquisition (equal), supervision (equal), writing – review and editing (equal). **Silvia Bittner Fialová:** conceptualization (equal), data curation (equal), funding acquisition (equal), investigation (equal), methodology (equal), project administration (equal), resources (equal), supervision (equal), visualization (equal), writing – original draft (equal), writing – review and editing (equal).

## Funding

This work was supported by Vedecká Grantová Agentúra MŠVVaŠ SR a SAV, VEGA 1/0170/24, VEGA 1/0226/22.

## Consent

All the authors involved in this manuscript give the consent for publication.

## Conflicts of Interest

The authors declare no conflicts of interest.

## Supporting information


**Figure S1:** High‐performance liquid chromatography (HPLC‐DAD) profile of oregano leaves (green) and rhizomes (brown) water extracts. Detection at λ = 280 nm. The position of compounds is given in Table 2 of the manuscript.
**Figure S2:** Heat flow diagrams displaying the metabolic activity of MRSA (on left) and MSSA (on right) in BHI broth without treatment (negative conrol) and impacted by oxacillin (positive control) 1 μg/mL (MRSA) and 0.25 μg/mL (MSSA).
**Figure S3:** Dose–response curve of HaCaT cells treated with Irinotecan for 72 h. Cell viability was assessed by MTT assay and expressed relative to the untreated control. The X‐axis represents log₁₀ [irinotecan hydrochloride] (μM), and the Y‐axis shows relative cell viability (% of control). Data are presented as mean ± SD of three independent replicates (*n* = 3). The half‐maximal inhibitory concentration (IC₅₀) was calculated using nonlinear regression in GraphPad Prism.
**Table S1:** The tested bacterial collection strains.

## Data Availability

The data supporting the results reported in the presented manuscript are available at the Department of Pharmacognosy and Botany, Faculty of Pharmacy Comenius University Bratislava, Slovakia (Silvia Bittner Fialová, fialova@fpharm.uniba.sk).
