# Supplementary material for: Bioactive Potential of Origanum vulgare Rhizomes: Phenolic Composition, Antioxidant, Antibacterial, and Cytotoxicity Profiles
Source: Food Sci Nutr. 2026 Jan 9;14(1):e71413. doi: 10.1002/fsn3.71413 (PMC12784173; doi:10.1002/fsn3.71413)
Supplement: Supplementary file 1 — Figure S1: High‐performance liquid chromatography (HPLC‐DAD) profile of oregano leaves (green) and rhizomes (brown) water extracts. Detection at λ = 280 nm. The position of compounds is given in Table 2 of the manuscript. Figure S2: Heat flow diagrams displaying the metabolic activity of MRSA (on left) and MSSA (on right) in BHI broth without treatment (negative conrol) and impacted by oxacillin (positive control) 1 μg/mL (MRSA) and 0.25 μg/mL (MSSA). Figure S3: Dose–response curve of HaCaT cells treated with Irinotecan for 72 h. Cell viability was assessed by MTT assay and expressed relative to the untreated control. The X‐axis represents log₁₀ [irinotecan hydrochloride] (μM), and the Y‐axis shows relative cell viability (% of control). Data are presented as mean ± SD of three independent replicates (n = 3). The half‐maximal inhibitory concentration (IC₅₀) was calculated using nonlinear regression in GraphPad Prism. Table S1: The tested bacterial collection strains. [file FSN3-14-e71413-s001.docx]

**Supporting Information**

**Bioactive Potential of *Origanum vulgare* Rhizomes: Phenolic Composition, Antioxidant, Antibacterial, and Cytotoxicity Profiles** Elena Kurin^1^, Kamila Dokupilová^1^, Ema Kostovčíková^1^, Lívia Slobodníková^2^, Eva Drobná^3^, Iveta Čičová^4^, Veronika Brindza Lachová^1^, Jana Sabová^5^, Peter Gál^5,6^, Milan Nagy^1^, Pavel Mučaji^1^, Silvia Bittner Fialová^1,*^

^1^Department of Pharmacognosy and Botany, Faculty of Pharmacy, Comenius University Bratislava, Odbojárov 10, 832 32 Bratislava, Slovakia

^2^Institute of Microbiology, Faculty of Medicine, Comenius University Bratislava, and the University Hospital in Bratislava, Sasinkova 4, 811 08 Bratislava, Slovakia

^3^Department of Cell and Molecular Biology of Drugs, Faculty of Pharmacy, Comenius University in Bratislava, Kalinčiakova 8, 832 32 Bratislava, Slovakia.

^4^National Agricultural and Food Centre, Research Institute of Plant Production, 921 01 Piešťany, Slovakia

^5^Department of Pharmacology, Faculty of Medicine, Pavol Jozef Šafárik University in Košice, Trieda SNP 1, 040 11 Košice, Slovakia

^6^Prague Burn Centre, Third Faculty of Medicine and University Hospital Královské Vinohrady, Šrobárova 50, 130 00 Prague, Czech Republic;

*Corresponding author. Email address: [fialova@fpharm.uniba.sk](mailto:fialova@fpharm.uniba.sk)

Table of Contents

[MATERIALS AND METHODS 2](#_Toc216850676)

[Extract preparation 3](#_Toc216850677)

[DPPH Radical Scavenging Assay 3](#_Toc216850678)

[ABTS Radical Scavenging Assay 3](#_Toc216850679)

[H_2_O_2_ Scavenging Assay 4](#_Toc216850680)

[Detection of Intracellular Oxidative Stress 4](#_Toc216850681)

[Cell Viability by MTT Assay 4](#_Toc216850682)

[Broth Microdilution Method of Antimicrobial Activity Testing 5](#_Toc216850683)

[**Table S1.** The tested bacterial collection strains. 5](#_Toc216850684)

[Microcalorimetry Method of Antimicrobial Activity Testing 6](#_Toc216850685)

[LC-MS-DAD 6](#_Toc216850686)

[Statistical analysis 7](#_Toc216850687)

[RESULTS 8](#_Toc216850688)

[**Figure S1.** High-performance liquid chromatography (HPLC-DAD) profile of oregano leaves (green) and rhizomes (brown) water extracts. Detection at λ = 280 nm. The position of compounds is given in Table 2 of the manuscript. 8](#_Toc216850689)

[**Figure S2.** Heat flow diagrams displaying the metabolic activity of MRSA (on left) and MSSA (on right) in BHI broth without treatment (negative conrol) and impacted by oxacillin (positive control) 1μg/mL (MRSA) and 0.25μg/mL (MSSA). 9](#_Toc216850690)

[**Figure S3.** Dose–response curve of HaCaT cells treated with Irinotecan for 72 h. Cell viability was assessed by MTT assay and expressed relative to the untreated control. The X-axis represents log₁₀ [irinotecan hydrochloride] (µM), and the Y-axis shows relative cell viability (% of control). Data are presented as mean ± SD of three independent replicates (n = 3). The half-maximal inhibitory concentration (IC₅₀) was calculated using nonlinear regression in GraphPad Prism. 10](#_Toc216850691)

##

# MATERIALS AND METHODS

Chemicals: 2,2-diphenyl-1-picrylhydrazyl radical (Sigma-Aldrich, St. Louis, MO, USA); rosmarinic acid (purity ≥ 95.5, Sigma-Aldrich, St. Louis, MO, USA); 2,2′-azino-bis(3-ethylbenzothiazoline-6-sulfonic acid (purity ≥ 98%, Sigma-Aldrich, St. Louis, MO, USA); potassium persulfate (purity ≥ 99.0, Sigma-Aldrich, St. Louis, MO, USA); ferrous ammonium sulphate (purity ≥ 99.0, Sigma-Aldrich, St. Louis, MO, USA); 1,10-phenanthroline (purity ≥ 99.0, Sigma-Aldrich, St. Louis, MO, USA). Dichlorodihydrofluorescein diacetate (purity ≥ 97%, Sigma-Aldrich, St. Louis, MO, USA); Dulbecco’s Modified Eagle Medium (Sigma-Aldrich, St. Louis, MO, USA); Fetal Bovine Serum (Sigma-Aldrich, St. Louis, MO, USA); streptomycin (Sigma-Aldrich, St. Louis, MO, USA); penicillin (Sigma-Aldrich, St. Louis, MO, USA); hydrogen peroxide (Sigma-Aldrich, St. Louis, MO, USA); MTT (3-(4,5-dimethyl-2-thiazolyl)-2,5-diphenyl-2*H*-tetrazolium bromide) (Sigma-Aldrich, Saint-Louis, USA). Aqua Pro Injectione Braun (B.Braun Melsungen AG, Melsungen, Germany), Millex – HA Filter Unit (Merck Millipore Ltd., Ireland); Mueller-Hinton Broth (OXOID Ltd., Basingstoke, Hampshire, UK); Sabouraud dextrose broth (OXOID, Basingstoke, Hampshire, UK); Skim Milk (Oxoid, Basingstoke, Hampshire, UK); Sabouraud agar (OXOID, Basingstoke, Hampshire, UK). NIH-3T3 cells (mouse embryonic fibroblasts), all used for the experiments were obtained from the Department of Pharmacology and Toxicology, Faculty of Pharmacy, Comenius University in Bratislava, Slovakia. HaCaT cells (human epidermal keratinocytes) (Cell Lines Service, Eppelheim, Germany). *Staphylococcus aureus* CCM 4223*, Staphylococcus aureus* CCM 4750*, Enterococcus faecalis* CCM 4224*, Pseudomonas aeruginosa* CCM 3955*, Escherichia coli* CCM 3954*, Klebsiella pneumoniae* CCM 4415, *Proteus mirabilis* CCM 7188, *Candida albicans* CCM 8621 (Czech Collection of Microorganisms, Brno, Czech Republic). 96-well Greiner UV star microplates (Greiner-Bio One GmbH, Frickenhausen, Germany); Infinite M200 microplate reader (Tecan AG, Grӧdig, Austria), black 96-well plates (Sarstedt, Nümbrecht, Germany); U-shaped, sterile 96-well microtiter plates (Roll s.a.s., Piove di Sacco, Italy); Analytical Balance (Electronic Balance, A&D Company, Limited, Japan). Methanol (LCMS grade, Merck KgaA, Darmstadt, Germany), water (LCMS grade, Merck KgaA, Darmstadt, Germany), acetonitrile (LCMS grade, Merck KgaA, Darmstadt, Germany), ammonium hydrogen carbonate (LC-MS LiChropur™, Merck KgaA, Darmstadt, Germany), formic acid 98% – 100% (LC-MS LiChropur™, Merck KgaA, Darmstadt, Germany), luteolin (primary RS, purity > 95%, Sigma Aldrich St. Louis, MO, USA), luteolin-7-*O*-diglucuronide (phyproof® RS, purity e (phyproPhytoLab GmbH & Co. KG, Germany) caffeic acid (purity Sigma Aldrich St. Louis, MO, USA), luteolin-7-, Germanrmant, Germant, German -MS armstadt, Germany), water r Lithospermic acid (phyproof® RS, purity ofid (phyPhytoLab GmbH & Co. KG, Germany), salvianolic acid A (analytical standard, purity ≥ 95.0%, Supelco® Sigma Aldrich St. Louis, MO, USA), irinotecan hydrochloride (purity ≥97%, Sigma Aldrich St. Louis, MO, USA).

## Extract preparation

10 g of oregano leaves or rhizomes (*Origanum vulgare* L.), respectively, were boiled for 5 minutes with 100 mL of hot deionized water (Direct-Q 8 UV Lab Water system, Millipore Corporation, USA) and cooled at room temperature for 45 min. The obtained extracts were filtered, frozen, and subjected to lyophilization following the manufacturer's instructions (SCANVAC CoolSafe™, LaboGene™, Lynge, Denmark). The lyophilization process was conducted at -53 °C and 0.043 Pa.

## DPPH Radical Scavenging Assay

The radical solution was prepared by dissolving 4.4 mg of DPPH^•^ in 200 mL of distilled methanol. The lyophilizates were dissolved in deionized water to various concentrations, while rosmarinic acid (RA), a positive control, was dissolved in distilled ethanol. Here, 25 μL of samples and 225 μL of DPPH^•^ solution were added to 96-well Greiner UV star microplates and mixed. The absorbance was measured after 30 minutes using an Infinite M200 microplate reader and compared with a blank. The antioxidant activities of lyophilizates were tested in quadruplicate and compared with the activity of RA. The percentage of radical inhibition was calculated using the following equation:

% = (A_blank_ – A_sample_)/A_blank_ x 100

where A_blank_ is blank (without sample).

## ABTS Radical Scavenging Assay

ABTS^•+^ was produced *via* the reaction of 7 mM water solution of ABTS with 2.45 mM water solution of potassium persulfate (1:1, *V*/*V*) and stored at the room temperature in dark for 16–24 hours before use. After that, 1.1 mL of the ABTS^•+^ solution was diluted with distilled ethanol to obtain the final volume of 50 mL. Oregano lyophilizates were dissolved in deionized water, while RA, a positive control, was dissolved in distilled ethanol to various concentrations. Here, 2.5 μL of samples and 247.5 μL of diluted ABTS^•+^ solution were added to 96-well Greiner UV star microplates and mixed. Absorbance was measured after 6 min using an Infinite M200 microplate reader and compared to the blank. The antioxidant activities measurements of lyophilizates were performed in quadruplicate and compared with the activity of RA. The percentage of radical inhibition was calculated using the following equation:

% = (A_blank_ – A_sample_)/A_blank_ x 100

where, A_blank_ is blank (without sample).

## H_2_O_2_ Scavenging Assay

Lyophilizates were dissolved in deionized water to various concentrations, while rosmarinic acid (RA), a positive control, was dissolved in distilled ethanol. Samples (500 µL) of different concentrations were mixed with 83.3 µL of ferrous ammonium sulphate (1 mM) and 20.8 μL of H_2_O_2_ (5 mM) and incubated in the dark for 5 minutes. After that, 500 µL of 1,10-phenanthroline (1 mM) was added to mixtures. After 10 minutes, 250 µL of mixtures were added to 96-well Greiner UV star microplates and absorbance was measured using an Infinite M200 microplate reader. The antioxidant activities of lyophilizates were tested in quadruplicate and compared with the activity of RA. The percentage scavenging of H_2_O_2_ generation was calculated using the following equation:

% = (A_sample_ – A_0_)/(A_max_ – A_0_) x 100

where, A_0_ is blank (without sample) and A_max_ is tris-1,10-phenantroline iron(II) complex (without sample and without H_2_O_2_).

## Detection of Intracellular Oxidative Stress

The reactive oxygen species (ROS) generation was measured using the fluorescence-based indicator dichlorodihydrofluorescein diacetate (DCFH-DA). Mouse embryonic fibroblasts (NIH-3T3 cells) were used for the experiment. The cells were grown in Dulbecco's Modified Eagle Medium (DMEM) supplemented with 10% Fetal Bovine Serum (FBS), 100 µg/mL streptomycin, and 100 IU/mL penicillin at 37 °C in a humidified atmosphere with 5% CO_2_. The cells were passaged approximately twice a week. The cells were seeded in the black 96-well plates at 15,000 cells/100 µL/well. After 24-hours incubation, the medium was removed and replaced by the serum-free medium. After 1-hour incubation, 5 µL of the tested oregano lyophilizates and RA, dissolved in the serum-free medium in various concentrations, were added. After 1 hour, DCFH-DA was added (10 µM in final concentration) and after 15 minutes, H_2_O_2_ was added (100 µM in final concentration). The intracellular fluorescence of dichlorodihydrofluorescein (DCF) was measured by excitation and emission at 480/530 nm after 15 minutes and compared to a blank in an Infinite M200 microplate reader. All measurements were performed in quadruplicate and compared with RA, which was used as a positive control. The percentage of radical inhibition was calculated using the following equation:

% = (A_blank_ – A_sample_)/A_blank_ x 100

where, A_blank_ is blank (without sample).

## Cell Viability by MTT Assay

Human epidermal keratinocytes (HaCaT cells) obtained from CLS Cell Lines Service (Eppelheim, Germany) were seeded (6000 cells/well) in a 96-well plate in culture medium with 10% FBS. After 24 hours the medium was replaced with complete medium containing different concentrations of oregano rhizomes or leaves lyophilizates, respectively. Following 72 hours incubation, cells were treated with MTT solution in final concentration 0.5 mg/mL for 2 hours at 37°C. Thereafter the medium was aspirated, and formazan crystals in each well were dissolved in 100 µL DMSO. Absorbance at 570 nm was measured by Infinite M200 microplate reader. Untreated cells were considered as a control. The absorbance of control was taken as 100%, and the results were expressed as a percentage of the control.

## Broth Microdilution Method of Antimicrobial Activity Testing

The MIC values were detected by broth microdilution test in the sterile U-shaped 96-well microtiter plates. The tested samples were added in 100 µL volumes to the starting wells and mixed with 100 µL volumes of double-concentrated antibiotic susceptibility test medium (Mueller-Hinton broth; OXOID, Basingstoke, Hampshire, UK) to obtain a concentration of 10 mg/mL. In the following wells, containing 100 µL volumes of Mueller-Hinton broth, serial geometric dilutions were prepared to reach the concentration range from 10 to 0.625 mg/mL. Bacterial inoculae were prepared from cultures (Table S1) grown overnight on blood agar. Well-isolated colonies were suspended in sterile physiologic solution and adjusted to 5 × 10^6^ CFU/mL. From this suspension, 10 µL volumes were added to each well, except for the sterility control wells (the negative controls) and incubated for 24 hours at 35°C. Wells with bacteria in the medium free of antimicrobial agents were used as growth control (positive controls). The MICs were determined as the lowest concentrations of the oregano lyophilizate samples without visible growth of the tested bacteria. MBCs were determined by sub-culturing of 5 µL sample volumes from the wells without detected bacterial growth on agar medium free of antimicrobial agents. After overnight incubation at 35 °C, the MBCs were determined as the lowest concentration at which 99.9% of the tested bacterial inoculum was killed.

The oregano samples were prepared as described in the antibacterial activity testing, but they were mixed with double concentrated Sabouraud dextrose broth to achieve the first tested dilution of 10 mg/mL. Sabouraud dextrose broth was used to prepare the rest of the dilutions. The yeast suspension was prepared from 48-hours culture on Sabouraud agar and adjusted in Sabouraud broth to achieve 5.10^5^ CFU/mL. This suspension was inoculated in 10 µL volumes to the microtiter plate wells. The plates were incubated aerobically at 35°C for 48 hours. Wells without yeasts and without tested lyophilizates were used as negative and positive controls. The MIC values and the minimal fungicidal concentrations (MFCs) were determined similarly to the antibacterial activity testing.

### **Table S1.** The tested bacterial collection strains.

| Bacterial Species | CCM / ATCC | Note |
| --- | --- | --- |
| *Staphylococcus aureus* | CCM 4223 / ATCC 29213 | MSSA; ATM susceptibility QC strain |
| *Staphylococcus aureus* | CCM 4750 / ATCC 43300 | MRSA; methicillin susceptibility reference strain |
| *Enterococcus faecalis* | CCM 4224 / ATCC 29212 | ATM susceptibility QC strain |
| *Pseudomonas aeruginosa* | CCM 3955 / ATCC 27853 | ATM susceptibility QC strain |
| *Escherichia coli* | CCM 3954 / ATCC 25922 | ATM susceptibility QC strain |
| *Klebsiella pneumoniae* | CCM 4415 / ATCC 10031 | ATM susceptibility QC strain |
| *Proteus mirabilis* | CCM 7188 / ATCC 29906 | Assays of ATM preservative QC |

MSSA – methicillin susceptible *Staphylococcus aureus*; ATM – antimicrobial; MRSA – methicillin-resistant *Staphylococcus aureus*, QC – quality control; CCM – Czech Collection of Microorganisms; ATCC – American Type Culture Collection.

## Microcalorimetry Method of Antimicrobial Activity Testing

For this method, we used two bacterial strains: MSSA and MRSA, listed in Tab. 1. The samples were prepared as described above and diluted using BHI (Brain-Heart Infusion, Millipore, Merck, Darmstadt, Germany) broth by serial geometric dilutions to reach the concentration range from 5 to 0.625 mg/mL for OVL and 5 to 0.500 mg/mL for OVR. 100 μL of the sample at different concentrations and 100 μL of bacteria (10^5^ CFU/mL) were inoculated into the calVial inserts placed in 48-well calPlate™ and properly secured with lids. The plate was placed into the calorimetry instrument for 24 hours. The impact of oregano extracts on bacterial metabolic activity was expressed as Heat Flow (μW) *vs*. Time, with Total Heat and Time to Peak parameters calculated using CalView software (Symcel, Sweden) and MS Excel (Microsoft, USA).

All tests were performed in three independent runs to exclude the possible excessive values due to permissible measurement error.

## LC-MS-DAD

Deionized water (adjusted to pH 3.1 with HCOOH/NH4HCO3) and acetonitrile were used as mobile phases A and B, respectively. The following gradient program was used: 5% B (0 min), 20% B (20 min), 20% B (25 min), 40% B (40 min), 50% B (60 min), 65% B (65 min), 100% B (90 min), 100% B (95 min), 5% B (96 min) and 5% (115 min). The ESI ion source parameters were as follows: capillary voltage: 3.5 kV; nebulizer: 40 psi (N_2_); dry gas flow: 10 L/min (N2); and dry temperature: 300°C. The mass spectrometer was operated in an auto MS^2^ mode, where each negative ion MS scan (m/z 30–1200, average of four spectra) was followed by MS^2^ scans (m/z 30–1200, average of four spectra, isolation window of 4 amu, collision energy 25 eV) of the two most intense precursor ions. Ions were excluded from analyses for 0.5 min after two MS^2^ spectra had been acquired. Nitrogen was used as the collision gas. Phenolic compounds were identified by measuring accurate m/z values and comparing measured UV and mass spectra with literature and authentic standards (when available).

The quantification of phenolic compounds in both oregano extracts was performed using external standards rosmarinic acid (at λ = 280 nm and 320 nm, respectively) and luteolin (at λ = 360 nm). Their calibration curves were obtained by injection of known concentrations (5–100 ppm). Both standards showed good linearity (r^2^ values shown below). The examinations were performed in triplicate. The quantitative results were calculated from calibration curves, expressed as mean values and standard deviation (SD).

## Statistical analysis

The experimental results were statistically analysed using GraphPad Prism version 5.00 for Windows, GraphPad Software, San Diego, California, USA. Data were expressed as mean ± standard deviation (SD) of triplicate/quadruplicate measurements and were analysed by Pearson’s two-tail correlation test. P ≤ 0.05 were considered as significant. Statistical changes in oregano parts were analyses by paired, two-tail Student's t-test or by ANOVA with Bonferroni post hoc test. P ≤ 0.05 were considered as significant.

# RESULTS

1

2

3

4

5

6

7

8

9

10

11

12

13

14

### **Figure S1.** High-performance liquid chromatography (HPLC-DAD) profile of oregano leaves (green) and rhizomes (brown) water extracts. Detection at λ = 280 nm. The position of compounds is given in Table 2 of the manuscript.


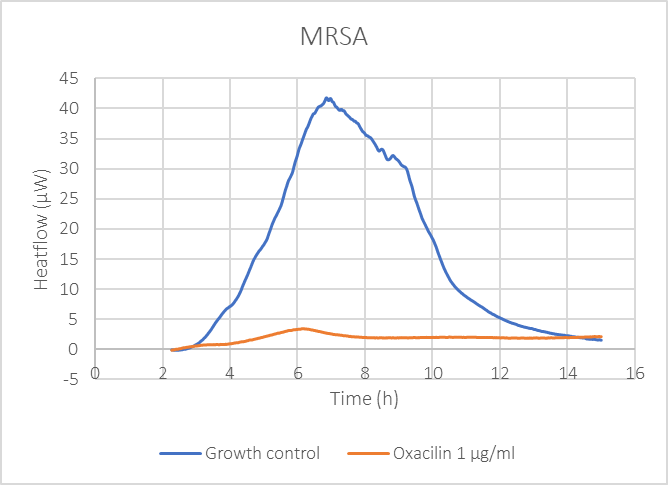

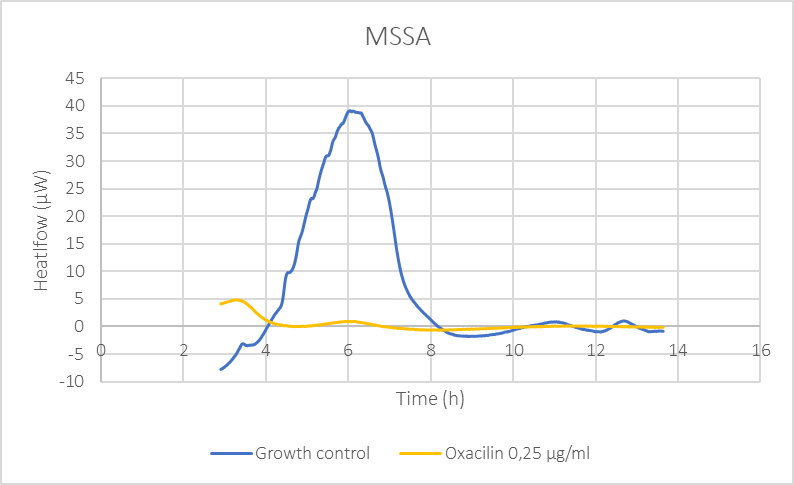


## **Figure S2.** Heat flow diagrams displaying the metabolic activity of MRSA (on left) and MSSA (on right) in BHI broth without treatment (negative conrol) and impacted by oxacillin (positive control) 1μg/mL (MRSA) and 0.25μg/mL (MSSA).


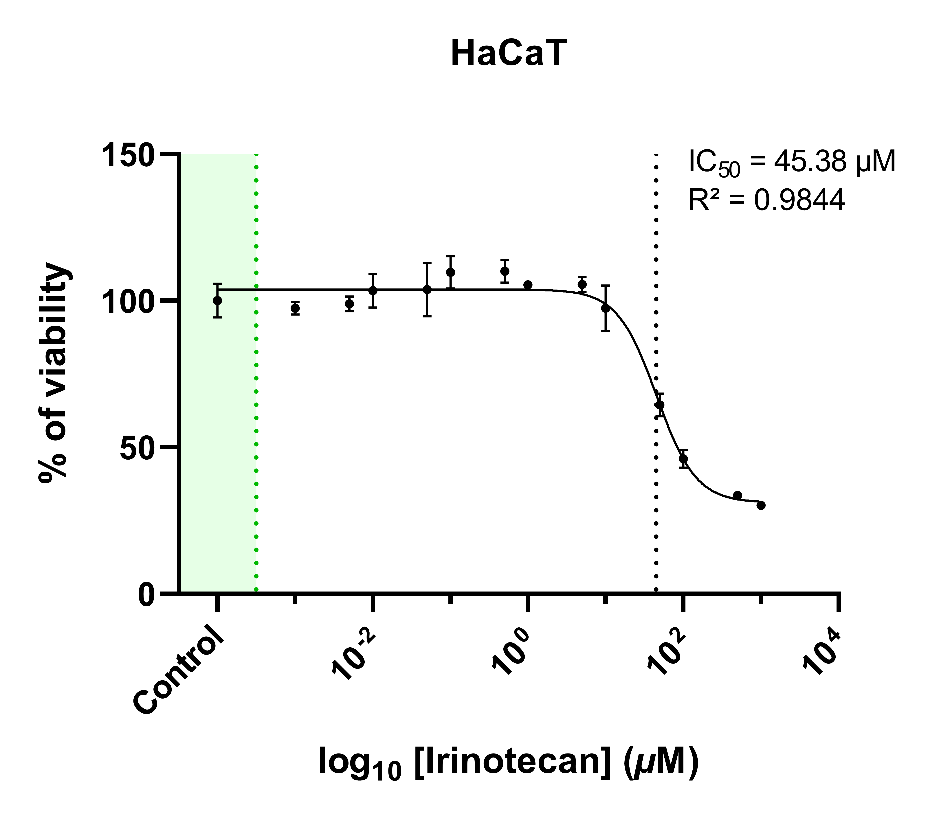


## **Figure S3.** Dose–response curve of HaCaT cells treated with Irinotecan for 72 h. Cell viability was assessed by MTT assay and expressed relative to the untreated control. The X-axis represents log₁₀ [irinotecan hydrochloride] (µM), and the Y-axis shows relative cell viability (% of control). Data are presented as mean ± SD of three independent replicates (n = 3). The half-maximal inhibitory concentration (IC₅₀) was calculated using nonlinear regression in GraphPad Prism.
